# Not just fat: investigating the proteome of cetacean blubber tissue

**DOI:** 10.1093/conphys/coy003

**Published:** 2018-02-16

**Authors:** Joanna L Kershaw, Catherine H Botting, Andrew Brownlow, Ailsa J Hall

**Affiliations:** 1 Sea Mammal Research Unit, Scottish Oceans Institute, University of St Andrews, St Andrews KY16 8LB, UK; 2 Biomedical Sciences Research Complex, School of Chemistry, Biomolecular Sciences Building Annexe, North Haugh, University of St Andrews, St Andrews KY16 9ST, UK; 3 Scottish Marine Animals Strandings Scheme, SAC Consulting Veterinary Services, Inverness IV2 5NA, UK

**Keywords:** Adipose tissue, biomarkers, marine mammals, metabolism, proteomics

## Abstract

Mammalian adipose tissue is increasingly being recognized as an endocrine organ involved in the regulation of a number of metabolic processes and pathways. It responds to signals from different hormone systems and the central nervous system, and expresses a variety of protein factors with important paracrine and endocrine functions. This study presents a first step towards the systematic analysis of the protein content of cetacean adipose tissue, the blubber, in order to investigate the kinds of proteins present and their relative abundance. Full depth blubber subsamples were collected from dead-stranded harbour porpoises (*Phocoena phocoena)* (*n* = 21). Three total protein extraction methods were trialled, and the highest total protein yields with the lowest extraction variability were achieved using a RIPA cell lysis and extraction buffer based protocol. Extracted proteins were separated using 1D Sodium Dodecyl Sulphate Polyacrylamide Gel Electrophoresis (SDS-PAGE), and identified using nanoflow Liquid Chromatography Electrospray Ionization in tandem with Mass Spectrometry (nLC-ESI–MS/MS). A range of proteins were identified (*n* = 295) and classed into eight functional groups, the most abundant of which were involved in cell function and metabolism (45%), immune response and inflammation (15%) and lipid metabolism (11%). These proteins likely originate both from the various cell types within the blubber tissue itself, and from the circulation. They therefore have the potential to capture information on the cellular and physiological stresses experienced by individuals at the time of sampling. The importance of this proteomic approach is two-fold: Firstly, it could help to assign novel functions to marine mammal blubber in keeping with current understanding of the multi-functional role of adipose tissue in other mammals. Secondly, it could lead to the development of a suite of biomarkers to better monitor the physiological state and health of live individuals though remote blubber biopsy sampling.

## Teaser

The separation and identification of proteins from cetacean blubber tissue will help to establish a database of potential health markers in species with limited sampling opportunities, and to assign novel functional roles to blubber in keeping with current understanding of the multi-functional role of adipose tissue in other mammals.

## Introduction

The thick layer of subcutaneous adipose tissue in marine mammals is called blubber, and is known to be important for energy storage (Slip *et al.*, 1992; Koopman *et al.*, 2002) maintaining hydrodynamic shape (Koopman *et al.*, 2002; Hamilton *et al.*, 2004), controlling positive buoyancy (Pabst *et al.*, 1999; McLellan *et al.*, 2002), thermal insulation (Parry, 1949; Ryg *et al.*, 1993) and thermoregulation (Hashimoto *et al.*, 2015). Blubber tissue can typically make up anywhere between 15% and 55% of the body mass of cetacean species (Ryg *et al.* 1993; McLellan *et al.* 2002). To date, most studies in cetaceans have focused on the physical properties of this tissue (Ackman *et al.*, 1975; McClelland *et al.*, 2012), the lipid content (Aguilar and Borrell, 1990; Evans *et al.*, 2003; Gómez-Campos *et al.*, 2011), variation in fatty acid profiles (Olsen and Grahl-Nielsen, 2003), and the quantification of lipophilic steroid hormones (Kellar *et al.*, 2009; Trego *et al.*, 2013; Kershaw *et al.*, 2017) and contaminants (Torres *et al.*, 2015; Jepson *et al.*, 2016). Adipose tissue in other mammals is known to be highly metabolically active and is increasingly being recognized as an endocrine organ in its own right (Kershaw and Flier, 2004; Gimeno and Klaman, 2005). To date, however, the blubber’s potential role as an endocrine organ in terms of contributing to whole body metabolism has yet to be explored.

As well as the fully differentiated adipocytes themselves, mammalian adipose tissue is made up of numerous other cell types including fibroblasts, pre-adipocytes, macrophages and other immune cells, endothelial cells and smooth muscle cells, connective tissue matrix, nerve tissue and blood vessels (Gimeno and Klaman, 2005; Frayn *et al.*, 2003). These components function as a structured whole which is known to express and secrete a variety of bioactive peptides which include proteins, metabolites and hormones, and are collectively known as adipokines (Trayhurn and Wood, 2004). These can act at both a local and systemic level (Kershaw and Flier, 2004; Trayhurn and Wood, 2004), and are derived from both adipocyte and non-adipocyte fractions of the tissue (Gimeno and Klaman, 2005). In addition to these secreted signals, adipose tissue expresses numerous receptors that allow it to respond to signals from a variety of hormone systems as well as the central nervous system. Its functions are therefore regulated by multiple external influences, such as the rate of blood flow into the tissue and thus the delivery of substrates and hormones in the plasma, autonomic nervous system activity (Frayn *et al.* 2003), and localized signalling and feedback loops within the tissue itself (Frühbeck *et al.*, 2001). These networks of adipose tissue signalling pathways enable the organism to adapt to a wide range of different metabolic challenges including starvation, stress, infection and periods of energy excess (Frühbeck *et al.* 2001). Through this network, adipose tissue is integrally involved in coordinating a variety of biological processes including the regulation of appetite and energy balance, immune system function, insulin sensitivity, angiogenesis, inflammation and the acute-phase response, blood pressure, nutrient transport and lipid metabolism and haemostasis (Trayhurn and Wood, 2004).

It is therefore now clear that adipose tissue is a complex and highly active metabolic and endocrine organ with fat cells playing an active role in modulating their own metabolism (Frühbeck *et al.* 2001; Kershaw and Flier, 2004; Trayhurn and Wood, 2004). The presence and concentrations of certain proteins and their metabolites in the tissue as well as circulating concentrations in the blood stream can therefore provide information on various processes and metabolic challenges. For example, circulating concentrations of adipose-derived hormones, leptin and adiponectin, are positively and negatively correlated with adiposity respectively (for review see Gimeno and Klaman, 2005). More generally, protein screening and identification are commonly used as diagnostic markers in medicine to detect disease and perturbations to metabolic pathways (Steffen *et al.*, 2016). Thus, both adipokines and other metabolic factors within the tissue are of interest to understand whole tissue function, the regulation of whole body metabolism and overall systemic health.

We aimed to investigate whether cetacean blubber, as the main adipose tissue store in cetacean species, could show equivalent pleiotropic functions to the adipose tissue in terrestrial animals by starting to identify some of the main protein components in the tissue. To achieve this, we had two main aims; Firstly, to reliably extract and separate the protein components in cetacean blubber. Secondly, to identify these components and link their presence to potential blubber functions. This process was split into three main stages: Firstly, method development for the extraction and separation of total protein from cetacean blubber tissue for the first time. Secondly, the identification of the extracted proteins by mass spectrometric analysis of protein digests and searching against a protein database for matches to homologous proteins from other vertebrate species. Thirdly, an investigation of which types of proteins were present and most represented in the extracts to make inferences about potential blubber functions and links to metabolic state.

This work has important implications for remotely obtained blubber samples from live cetaceans as samples collected through dart biopsying of free-ranging individuals has become one of the most common methods for obtaining biological samples from these species (Hunt *et al.*, 2013). The accessibility of blubber for sampling, together with its key role in metabolic homoeostasis makes it a valuable tissue for conservation physiologists. The separation and identification of proteins in blubber tissue are important steps in establishing a database of baseline, identifiable proteins and subsequently applying a proteomic approach to using various proteins as biomarkers of health and condition in these species with otherwise limited sampling opportunities from live animals. This information could therefore represent a valuable tool for conservation physiologists to assess health and responses to changing environments that have the potential for population level impacts.

## Methods

### Sample collection and preparation

Full-depth skin, blubber and underlying muscle samples were collected from dead harbour porpoise (*Phocoena phocoena)* by the Scottish Marine Animal Strandings Scheme (SMASS) between 2013 and 2015. Only freshly dead animals, specifically those that either stranded alive or had recently died, and thus showed no evidence of bloating and decomposition, were used in order to investigate blubber proteins that had not been subject to extensive degradation and metabolism after death. Samples showed no evidence of trauma or bruising as blood in the tissue would disproportionally represent proteins in circulation rather than proteins present in the blubber matrix itself. Samples were collected from the dorsal area immediately caudal to the dorsal fin. This site was chosen as this area is typically sampled through remote biopsy of free-ranging individuals and therefore has the most relevance for investigating the potential use of these total protein extraction methods on samples collected from live animals. A total of 24 samples were used from 21 individuals that were a mixture of both males (*n* = 10) and females (*n* = 11), and adults (*n* = 12) and juveniles (*n* = 9). The cause of death was determined following post-mortem examination, and individuals died as a result of acute trauma (entanglement in fishing gear, live standing, storm damage, dystocia and bottlenose dolphin or grey seal attacks) or chronic debilitation (starvation and infectious disease). For full details of the samples used for analysis see Table 1.

**Table 1:** Harbour porpoise blubber samples used for total protein extraction, separation and protein identification from gel bands excised following 1D SDS-PAGE

Processing method	Animal ID	Sex	Age class	Cause of death	Cause of death class	Gel bands
1	M343/13	F	J	Bycatch	Acute trauma	
	M396/13	F	A	Bycatch	Acute trauma	
	M134/14	F	A	Dystocia	Acute trauma	
	M055/14	M	A	Bycatch	Acute trauma	
	M307/14	M	A	Bottlenose dolphin attack	Acute trauma	
	M355/14	M	J	Bottlenose dolphin attack	Acute trauma	
	M061/15	F	J	Infectious disease	Chronic debilitation	
	M020/15	F	A	Infectious disease	Chronic debilitation	
2	M072/13	M	J	Live stranding	Acute trauma	2
	M018/13	M	A	Infectious disease	Chronic debilitation	
	M060/13	M	A	Bottlenose dolphin attack	Acute trauma	
	M040/14	M	J	Chronic entanglement	Chronic debilitation	
	M028/14	M	J	Bottlenose dolphin attack	Acute trauma	8
	M027/14	M	A	Storm damage	Acute trauma	
	M068/14	F	J	Starvation	Chronic debilitation	8
	M147/14	F	A	Infectious Disease	Chronic debilitation	
3	M343/13	F	J	Bycatch	Acute trauma	
	M315/13	F	A	Bottlenose dolphin attack	Acute trauma	
	M373/13	F	J	Bycatch	Acute trauma	
	M265/13	F	A	Infectious disease	Chronic debilitation	
	M134/14	F	A	Dystocia	Acute trauma	
	M307/14	M	A	Bottlenose dolphin attack	Acute trauma	14
	M131/15	M	A	Bottlenose dolphin attack	Acute trauma	4
	M144/15	F	J	Grey seal attack	Acute trauma	

Animal ID number is assigned by the SMASS at the time of sampling and sex (Female, Male), age class (Adult, Juvenile) and cause of death are assigned at necropsy. ‘Gel bands’ indicate the number of individual protein bands excised from various gels from five individuals that were analysed using nLC-ESI MS/MS.

Samples were collected and frozen in individual plastic vials at −20°C prior to analysis. For the total protein extraction, subsamples were taken on ice while the tissue was still frozen, and care was taken to remove all skin and muscle from the blubber. Full depth subsamples of each original sample were used for total protein extraction in order to investigate the proteins through all layers of the tissue.

### Total protein extraction methods

Protein extraction from a tissue typically involves three stages: (1) tissue and cell disruption through homogenization, (2) precipitation of the protein fraction of the homogenate into a pellet form and (3) resuspension of the protein pellet into solution for quantification and downstream applications.

In a comparative study of different detergent-free protein extraction protocols using these three stages, the most suitable method for the extraction of white adipose tissue proteins from a wide range of cellular and structural compartments was a de-lipidation protocol based on the Bligh and Dyer method (1959) (Sajic *et al.*, 2011). The optimal tissue and cell disruption part of the protocol described by Sajic and colleagues was replicated here using blubber samples collected from harbour porpoises. Then, two different protein precipitation methods were trialled. A protein precipitation method using a methanol–chloroform solution adapted by Friedman and colleagues (2009) for the recovery of proteins in dilute solution in the presence of detergents and lipids (Wessel and Flügge, 1984), was trialled first (Method 1). A second protein precipitation method using a trichloroacetic acid (TCA)–acetone solution was also trialled which aggressively removes non-protein compounds (Wu *et al.*, 2014) (Method 2). TCA is often used for precipitation as it is effective at low concentrations (Wu *et al.*, 2014). The sample volume therefore does not increase dramatically, and the protein concentration remains high which increases the efficiency of the precipitation (Wu *et al.*, 2014).

**Figure 1:**
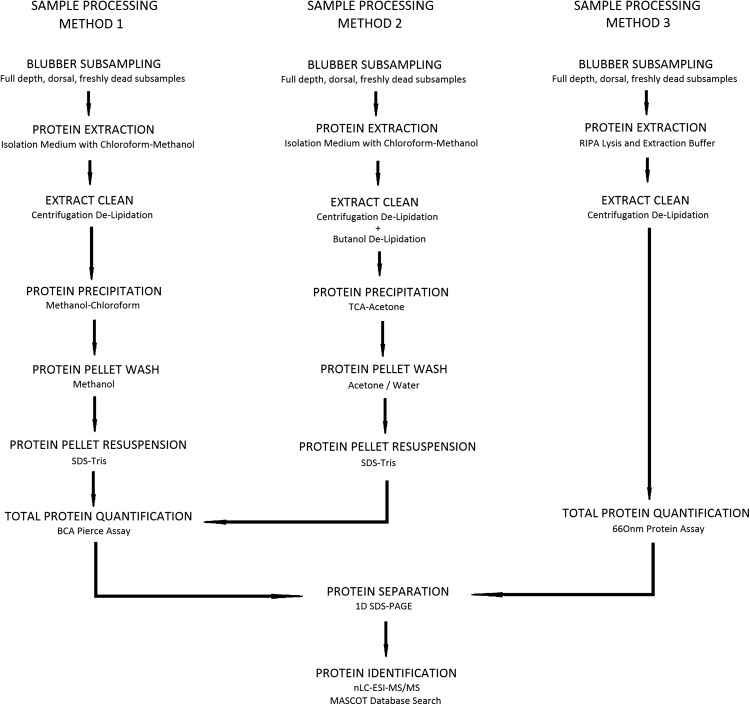
Workflow showing the sample processing methods for protein extraction, quantification, separation and identification. Sample processing Methods 1 and 2 involve various extract cleaning, protein precipitation and protein pellet wash stages. Sample processing Method 3 involves fewer processing stages. The extracts using all three methods were used for downstream analyses; protein quantification, separation and identification.

Finally, a simpler extraction method using Radioimmunoprecipitation Assay (RIPA) cell lysis and extraction buffer was trialled to assess if fewer sample processing stages, without the precipitation of protein into a pellet and resuspension into solution, results in a higher protein yield and less extraction variability (Method 3). RIPA cell lysis buffer is highly effective for protein extraction from a variety of cell types because it contains three non-ionic and ionic detergents. One disadvantage, however, is that this detergent formulation is incompatible with certain downstream applications compared to other lysis reagents, and there is no possibility to suspend the extracted protein in a different buffer for further analysis. For a summary of the three sample processing methods (Methods 1, 2 and 3) with their different stages see Fig. 1.

#### Method 1: Methanol–chloroform precipitation with methanol pellet wash

For full method details see the Supplementary materials, but briefly, 16 duplicate blubber subsamples, from eight individuals (Table 1), were weighed (0.4–0.6 g) and homogenized in isolation medium (50 mM Tris, 150 mM NaCl, 0.2 mM EDTA and 10 µg/ml protease inhibitors) and 1:2 chloroform/methanol. Samples were placed on ice and vortexed several times to ensure thorough mixing. Equal volumes of chloroform and deionised water were added. The sample was vortexed and centrifuged, and the upper, protein-containing phase and the protein disc were collected. Three volumes of water, four volumes of methanol and one volume of chloroform were added to the solution and vortexed. The mixture was centrifuged and the supernatant removed leaving only a protein pellet. Methanol was added to wash the pellet. The mixture was vortexed, then centrifuged, and the supernatant was removed and the protein pellet dried under nitrogen at room temperature. Care was taken not to over dry the pellet causing it to become flaky and stick to the centrifuge tube which reduces the resuspension efficiency. The pellet was resuspended in SDS/Tris (0.1% SDS in 40 mM Tris).

#### Method 2: TCA–acetone precipitation with acetone pellet wash

As above, 16 duplicate blubber subsamples, from eight different individuals (Table 1), were processed following the same protocol as previously described up to the collection of the upper protein-containing phase and the protein disc. 10% TCA in acetone was added to the sample, mixed and frozen overnight at −20°C. The sample was allowed to warm to room temperature and then centrifuged. The protein pellet was washed twice with ice cold acetone. Any remaining acetone following the final wash was evaporated under nitrogen at room temperature. Again, care was taken not to over dry the protein pellet, and it was resuspended in SDS/Tris. For full method details see the Supplementary materials. Of the two protein precipitation methods, using a TCA–acetone solution showed lowest total protein inter-assay variability (Table 2). For this reason, alterations were made to improve this protocol in an attempt to, firstly, further clean the extract, and secondly, to improve protein pellet resuspension by altering the pellet washing procedure.

**Table 2:** Summary of the total protein assay results for the three different extraction methods and alterations

	Extraction Methods 1 and 2				Extraction Method 3
	Methanol–chloroform protein precipitation	TCA–acetone protein precipitation			RIPA cell lysis and extraction buffer
		Original: acetone wash	Alteration 1: butanol de-lipidation	Alteration 2: water wash	
Total protein assay	Pierce BCA	Pierce BCA	Pierce BCA	Pierce BCA	Pierce 660 nm
Minimum protein yield	14.4 µg/g	3.8 µg/g	7.3 µg/g	7.7 µg/g	3101.8 µg/g
Maximum protein yield	361.6 µg/g	918.0 µg/g	158.8 µg/g	89.3 µg/g	5512.7 µg/g
Extraction CVs (^SD^/_mean_) × 100 duplicate protein extracts	28.4% (2.3–78.7%)	42.0% (6.7–90.0%)	50.6% (12.5–151.0%)	31.8% (2.1–68.8%)	21.9% (2.8–39.8%)
Inter-assay CVs (^SD^/_mean_) × 100 different total protein plates	86.5% (47.8–133.1%)	42.5% (0.5–141.4%)	31.6% (4.1–103.9%)	23.4% (3.31–69.5%)	15.8% (1.5–64.9%)
Intra-assay CVs (^SD^/_mean_) × 100 same total protein plate	0.7% (0.1–1.3%)	2.0% (1.2–3.3%)	28.7% (4.7–100.0%)	19.3% (2.5–105.6%)	4.5% (3.6–5.5%)

CV, coefficient of variation; SD, standard deviation.

##### Alteration 1: TCA–acetone precipitation with butanol de-lipidation and acetone pellet wash

As cetacean blubber tissue has such a high lipid content, an extra butanol de-lipidation step was added before the precipitation of the protein pellet in an attempt to further ‘clean’ the extract (Zhao and Xu, 2010). Another 16 duplicate blubber subsamples were taken from the same eight individuals used for the TCA–acetone precipitation protocol for direct comparison. Following the collection of the upper protein-containing phase and protein disc (after homogenization and centrifugation), butanol was added to the extract. This was then centrifuged and the upper phase containing any remaining lipids was discarded. 10% TCA in acetone was added to the lower phase, and the precipitation and pellet wash continued as described above. The final protein pellet was resuspended in SDS/Tris.

##### Alteration 2: TCA–acetone precipitation with water wash

Poor resuspension of the protein pellet in the SDS–Tris could result in underestimates of measured total protein content and inconsistencies, if some pellets resuspend less efficiently than others. The effect of a different pellet wash was tested by using deionised water instead of acetone for the final wash stage in an attempt not to dehydrate the pellet and therefore make its resuspension back into solution more efficient. Again, 16 duplicate blubber subsamples were taken from the same eight individuals used for the TCA–acetone precipitation protocol for direct comparison. The same extraction and precipitation protocol was followed, then upon the precipitation of the proteins, the pellet was washed twice by adding deionised water, vortexed, centrifuged and the supernatant was discarded. Care was taken not to disturb the washed pellet that was then resuspended in SDS/Tris.

#### Method 3—RIPA lysis and extraction buffer

Finally, 16 duplicate blubber samples, from another eight individuals (Table 1), were subsampled on ice while frozen and weighed (0.1 ± 0.01 g). Briefly, the frozen tissue was placed on ice in low protein binding micro-centrifuge tubes, with RIPA lysis and extraction buffer (Thermo Fisher Scientific) containing 2× concentration of protease inhibitors (Pierce Protease Inhibitor Mini Tablets). The samples were homogenized and replaced on ice before centrifugation. The protein-containing infranatant was then removed and placed into a clean, low protein binding micro-centrifuge tube. The protein fraction was centrifuged, as before, as a second de-lipidation step, and replaced on ice. Again, the infranatant was removed and placed in a clean, low protein binding micro-centrifuge tube and was used for analysis.

### Total protein quantification assays

Two different commercially available total protein quantification kits were used to measure the protein in the blubber extracts based on their compatibility with samples resuspended in either SDS/Tris or RIPA buffer. Firstly, a Pierce™ BCA Protein Assay Kit (23225, Thermo Scientific, Rockford, USA) was used to quantify the total protein in the extracts prepared using Methods 1 and 2 (Fig. 1). A series of dilutions of known concentrations of bovine serum albumin were used as a standard curve, and the microplate procedure was carried out following the kit instructions, measuring the absorbance due to formation of the bicinchoninic acid Cu^+^ complex at 562 nm. Each extract was assayed in duplicate on every plate, and each extract was assayed on three different plates in order to determine the inter-assay coefficient of variation (% CV). The average concentration measured across the three plates was used as the final estimated total protein content of a sample. Total protein concentrations are reported as microgram per wet weight of the sample. Secondly, a Pierce™ 660 nm Protein Assay (22662, Thermo Scientific, Rockford, USA) was used to quantify the total protein in the extracts prepared using Method 3. The blubber extracts were prepared following the assay instructions for cell lysates in RIPA buffer by adding Triton™ X-100. A standard curve was prepared as described above following the kit instructions for the microplate protocol, measuring absorbance at 660 nm. Each extract was assayed in duplicate and total protein concentrations are reported as μg per wet weight of the sample. Each extract was assayed on two different plates in order to determine the inter-assay % CV, and the average concentration measured across the two plates was used as the final estimated total protein content of a sample.

### 1D SDS-PAGE protein separation

The final stage of the method development was to optimize protein separation and visualization using 1D SDS-PAGE which separates denatured proteins based on their size. For full details see the Supplementary methods. Briefly, 4–12% NuPAGE Bis-Tris mini gels (8.0 cm × 8.0 cm × 1.0 mm) (NP0321BOX, Thermo Fisher Scientific, Paisley, UK) were used. The recommended sample and MES running buffers for denatured proteins as listed by the manufacturers, as well as the standard running conditions were used to run the gels. A wide protein range ladder, Invitrogen^TM^ Novex^TM^ Mark 12^TM^ Unstained Standard (LC5677, Fisher Scientific, USA) was run on each gel. The blubber extracts resuspended in SDS/Tris were run undiluted on the gels, while the extracts in RIPA buffer were run at ½ and ^1^/_3_ dilutions for better band separation, as the protein content of these extracts was much higher. Upon completion of the run, the gels were stained in Bio-Safe Coomassie Stain (1610786, Bio-Rad, UK), and were then rinsed and destained in deionised water for up to 48 h. The destained gels were photographed on a white background using a BioDoc-It™ Imaging System (Ultra-Violet Products Ltd, Cambridge, UK).

### Quality assurance/quality control

Total protein extraction method comparison studies use various approaches to assess the protein extraction efficiencies for particular tissues including total protein yield, distribution of molecular weights of extracted proteins separated using 1D SDS-PAGE, reproducibility of protein bands with minimal streaking and background using 1D SDS-PAGE, presence of specific protein markers from different cellular compartments using Western Blot techniques, presence/absence of individual protein spots using 2D SDS-PAGE, and reproducibility of protein spot patterns using 2D SDS-PAGE in both animal (Jiang *et al.*, 2007; Cilia *et al.*, 2009; Sajic *et al.*, 2011; Panchout *et al.*, 2013) and plant (Natarajan *et al.*, 2005; Sheoran *et al.*, 2009) studies. In keeping with such studies, here, the performance of the different extraction protocols in terms of their ability to efficiently and consistently extract protein from blubber tissue was assessed in four ways by (i) measuring total protein yield, (ii) measuring extraction variability between duplicate extracts of the same sample extracted and assayed in tandem, (iii) measuring assay variability in terms of the protein measured in the same sample over multiple assays and (iv) visually inspecting the molecular weight distribution, the number and reproducibility of protein bands separated by 1D SDS-PAGE.

**Figure 2:**
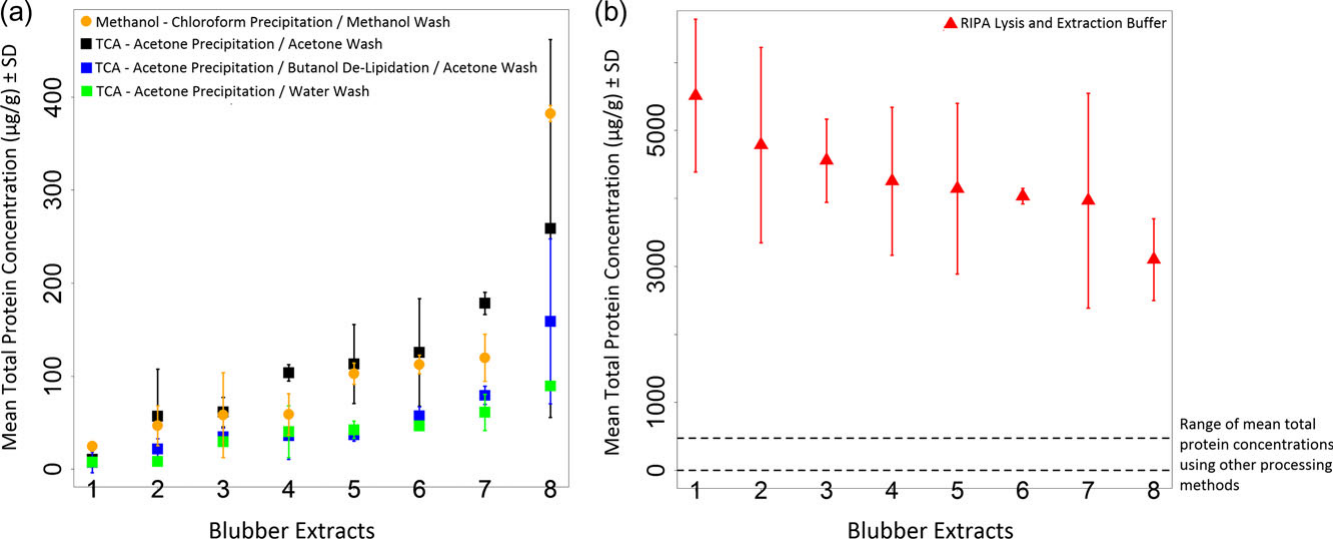
Total protein yield in blubber extracts using different sample processing Methods 1, 2 and 3. (**a**) Total protein concentrations measured in extracts processed using Method 1 and the three variations of Method 2. (**b**) Total protein concentrations measured in extracts processed using Method 3. Overall, the total protein yield was an order of magnitude higher using this method although there was still high extraction variability.

All statistical analyses were performed using the statistical package, R, version 3.1.3. A one way analysis of variance (ANOVA) was used to compare the mean protein yields of all extracts processed using the five different method variations (Method 1, three alterations of Method 2, and Method 3). The extraction variability for each sample extracted in duplicate was calculated as a % coefficient of variation, and again, a one-way ANOVA was used to compare between the mean % extraction variability across the five method variations (Table 2). The inter-assay coefficients of variation for samples assayed multiple times across different plates were also assessed using a one-way ANOVA to determine differences in the repeatability of the measurements of the same extracts (Table 2). Finally, the 1D SDS-PAGE gels were assessed for the range of molecular weights of the bands that were separated, the number of protein bands separated and the consistency with which these bands appeared in multiple extracts and across multiple gels (see Supplementary Results Figs 1 and 2 for comparative example 1D gel images of extracts processed using the different methods).

### Protein identification

A total of 36 protein bands were excised from five harbour porpoise individuals run on four different 1D SDS-PAGE gels, and were stored in individual micro-centrifuge tubes at 4–8°C for subsequent protein identification. The bands were from blubber extracts processed using Methods 2 and 3 (Table 1), and covered the full size range of separated proteins from the largest ones of more than 200 kDa, down to the smallest bands visible at ~10 kDa. In order to capture the full range of proteins that could be present in the tissue, bands were all of different molecular weights and were chosen from a mixture of males and females, and from adults and juveniles with varying causes of death (Table 1). The darkest stained bands (indicating highest protein concentration) were chosen which allowed the clear visual separation of the band on the gels. These were analysed using nanoflow Liquid Chromatography Electrospray Ionization in tandem with Mass Spectrometry (nLC-ESI MS/MS) of in-gel trypsin digests.

The excised gel band was cut into 1 mm cubes. These were then subjected to in-gel digestion, using a ProGest Investigator in-gel digestion robot (Genomic Solutions, Ann Arbor, MI) using standard protocols (Shevchenko *et al.*, 1996). Briefly the gel cubes were destained by washing with acetonitrile and subjected to reduction and alkylation before digestion with trypsin at 37°C. The peptides were extracted with 10% formic acid and concentrated down 20 times using a SpeedVac (ThermoSavant). The peptides were then injected on an Acclaim PepMap 100 C18 trap and an Acclaim PepMap RSLC C18 column (ThermoFisher Scientific) using a nanoLC Ultra 2D plus loading pump and nanoLC as-2 autosampler (Eksigent). The peptides were eluted with a gradient of increasing acetonitrile, containing 0.1% formic acid (5–40% acetonitrile in 6 min, 40–95% in a further 2.5 min, followed by 95% acetonitrile to clean the column, before re-equilibration to 5% acetonitrile). The eluate was sprayed into a TripleTOF 5600+ electrospray tandem mass spectrometer (Sciex, Foster City, CA) and analysed in Information Dependent Acquisition (IDA) mode, performing 250 ms of MS followed by 100 ms MSMS analyses on the 20 most intense peaks seen by MS The MS/MS data file generated via the ‘Create mgf file’ script in PeakView (Sciex) was analysed using the Mascot search algorithm (Matrix Science), against the NCBInr database (Apr and Oct 2015 and Aug 2016) with no species restriction (65 519 838 to 93 482 448 sequences), trypsin as the cleavage enzyme, and carbamidomethyl as a fixed modification of cysteines and methionine oxidation as a variable modification. The peptide mass tolerance was set to 20 ppm and the MSMS mass tolerance to ±0.05 Da.

A protein was accepted as identified if it had two or more peptides with Mascot Ion Scores above the Identity Threshold (*P* < 0.05), and, for those proteins identified by only two peptides, the MSMS spectral assignments fulfil the criteria described in Jonscher (2005). The sequences matched to homologous vertebrate proteins.

## Results

### Method development and optimization

There was considerable variation seen in both the total protein yields, and the extraction variability between duplicate subsamples (Fig. 2a and b). Of the two precipitation methods, the TCA–acetone precipitation method was chosen for further development. An extra butanol de-lipidation step and pellet wash with water were trialled in an attempt to further optimize the method in order to improve consistency. However, even with these changes, the total protein yield was still poor, and there were no statistically significant differences between the overall protein yield between the four precipitation methods (Method 1 and the 3 variations of Method 2) (ANOVA; df = 3, *F* = 2.33, *P* = 0.08) (Fig. 2a). The highest total protein yield, by at least an order of magnitude, was obtained using the third extraction method with RIPA cell lysis buffer which was significantly higher than all other extracts (ANOVA; df = 4, *F* = 280.1, *P* < 0.0001) (Fig. 2b). The extraction variability was high and was not statistically different between methods (ANOVA; df = 4, *F* = 1.13, *P* = 0.36), but extracts processed using Method 3 had a lower mean extraction variability and a smaller range across duplicate samples (Table 2).

There was a wide range of inter-assay variability across all samples, particularly for Methods 1 and 2, which were largely over the acceptable inter-assay % coefficient of variation threshold of 20%, based on general protocols for immunoassay validations (Grotjan and Keel, 1996) (Table 2). The extracts processed using Method 1 had significantly higher inter-assay CVs than the other methods (ANOVA; df = 4, *F* = 11.94, *P* < 0.001), while the others were not significantly different to each other. Three of the five extraction protocols generally gave low average intra-assay % CVs below the 10% threshold that was considered acceptable for three extracts assayed twice on each plate (Grotjan and Keel, 1996) (Table 2). As the total protein standard curves were all almost identical between different plates (data not shown), the high between and within assay variation measured here for the Pierce BCA assay is likely indicative that measurement error or artefacts due to the assay reagents and the assay process were not the cause of the variability. Instead, resuspension of the protein in solution was likely a problem for reliable and consistent measurement of the precipitated proteins.

### Protein identification

**Figure 3:**
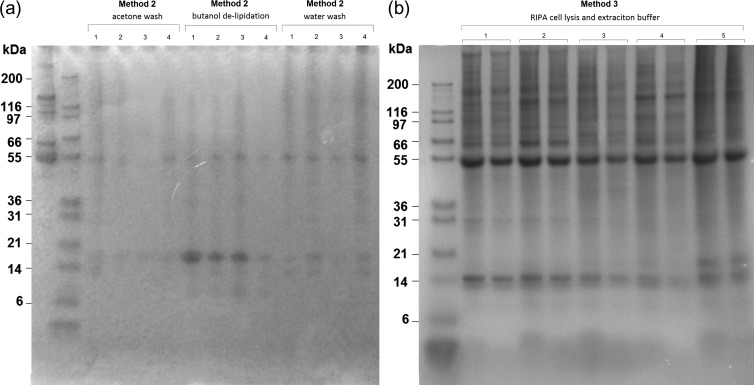
1D SDS-PAGE analysis of harbour porpoise blubber tissue extracts on 4–12% Bis-Tris gels stained with Bio-Safe Coomassie brilliant blue. (**a**) Protein extracts labelled 1–4 were extracted using TCA–acetone precipitation with an acetone wash, TCA–acetone precipitation with butanol de-lipidation, and TCA–acetone precipitation with a water wash. (**b**) Protein extracts labelled 1–5, extracted in RIPA cell lysis buffer, and each diluted ^1^/_2_ and ^1^/_3_.

A total of 295 proteins were identified across the 36 gel bands separated through 1D SDS-PAGE from five individuals. There was a wide range in molecular weights of the separated protein bands, with both more numerous and more consistent bands seen in the samples processed in RIPA cell lysis buffer (Fig. 3). (For more 1D gel example images see Supplementary Results, Figs 1 and 2.) Many of the proteins, and protein fragments were identified across multiple bands from the same gel, and therefore did not show clear clustering around their expected molecular weight range. This was possibly due to protein degradation and/or some proteins being more abundant than others. The identified proteins were grouped into general subclasses firstly, based on their type, and secondly based broadly on their function using data from UniProt (http://www.uniprot.org) and a literature search. This resulted in the identification of proteins belonging to 5 main types: enzymes (proteins involved in the catalysis of various processes), immune proteins (proteins involved in the regulation of immune system function and activation as well as inflammation), carrier proteins (proteins that bind other factors for extracellular or intracellular transport), structural proteins (proteins responsible for the maintenance of cell shape and integrity as well as the extracellular matrix) and regulatory proteins (any other proteins involved in the regulation of other cellular processes) were grouped together.

Proteins were then grouped into functional classes which resulted in the identification of eight main groups: amino acid metabolism, lipid metabolism, tissue structure, cell structure, glucose homoeostasis, biomolecule transport, immune response and inflammation and overall cell function and metabolism. As an indicator of relative abundance, the frequency with which each protein was identified across different gel bands and individuals was also recorded in order to identify the kinds of proteins that appeared to be most abundant. Over half of all proteins identified were only seen once across all 36 gel samples analysed, while less than 5% of the proteins were seen more than 10 times. The top six proteins were haemoglobin, immunoglobins, serum albumin, fatty acid-binding protein, myoglobin and annexins.

**Figure 4:**
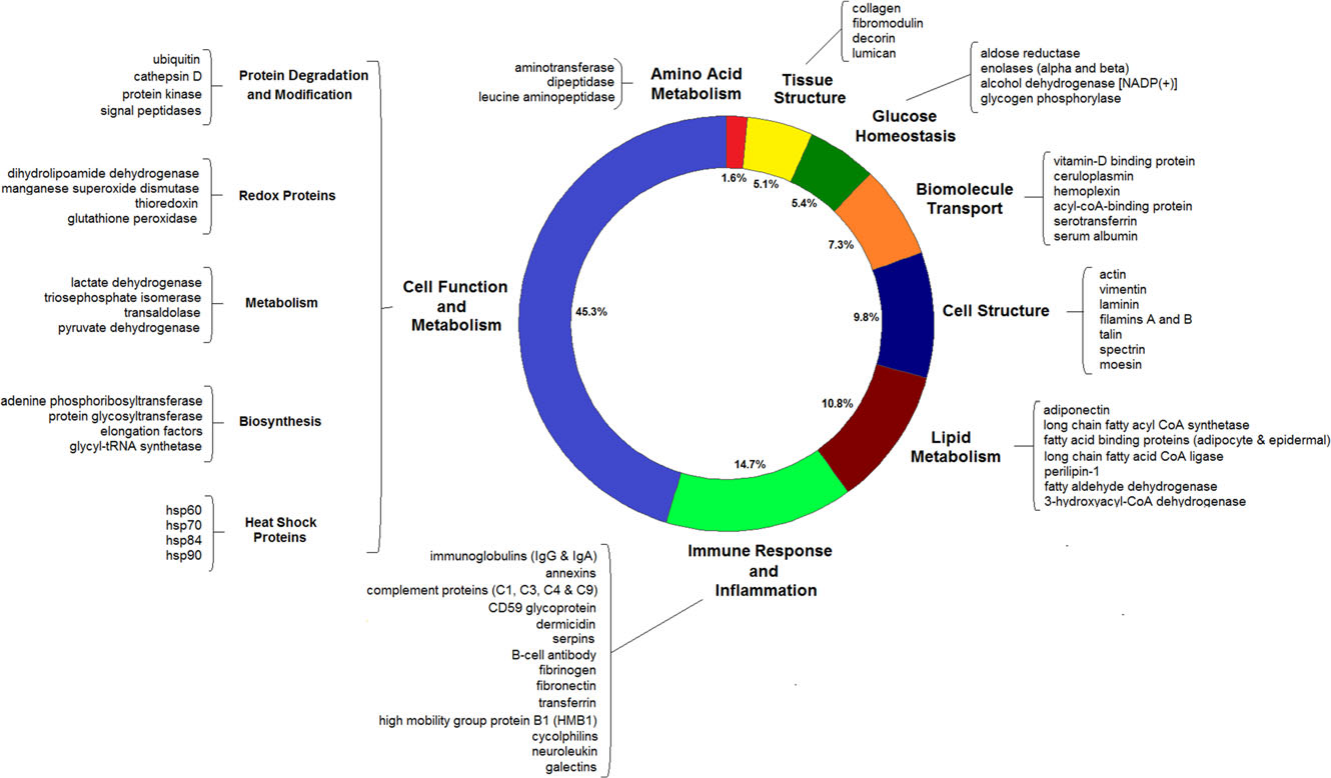
Protein components identified from the blubber extracts. The proportion of each functional group is indicated as well as examples of some of the most abundant and the most well-studied proteins that were identified. The most abundant functional group was proteins involved in cell function and metabolism followed by the immune response and inflammation.

Proteins involved in amino acid metabolism, tissue structure, glucose homoeostasis, biomolecule transport and cell structure were the least abundant of the protein functional groups, each making up less than 10% of all the identified proteins (Fig. 4). Proteins involved in lipid metabolism made up ~10% of the identified proteins and these included one of the adipokines, adiponectin, regulatory proteins including fatty acid-binding proteins and perilipin-1, as well as enzymes including 3-hydroxyacyl-CoA dehydrogenase and acetyl CoA synthetase for example (Fig. 4).

The second largest functional group were immune proteins. These were of varying sizes and functions, and made up ~15% of all identified proteins (Fig. 4). The immune proteins were dominated by two classes of immunoglobulin, IgA and IgG. Annexins and cyclophilins were also present, and are involved in the regulation of the inflammatory response (Bukrinsky, 2015; Sugimoto *et al.*, 2016). Other proteins included transferrin, fibrinogen and fibronectin that are involved in the acute-phase response (Kilicarslan *et al.*, 2013). Dermicidin has antimicrobial properties (Schittek *et al.*, 2001), and 4 complement proteins (C1, C3, C4 and C9) were identified that act as parts of the complement system which forms part of innate immunity (Nesargikar *et al.*, 2012). B-cell antibody and galectins expressed by immune cells were also identified (Fig. 4).

Finally, the largest functional group were proteins involved in general cell function and metabolism, and made up ~45% of the proteins identified. These were proteins involved in a range of different processes including protein degradation and modification, biosynthesis, metabolic pathways (e.g. glycolysis), redox proteins, heat shock proteins, signal transduction, vesicle trafficking, cell cycle regulation and protein chaperones, to name just a few (Fig. 4). For full details, see Supplementary Table 1.

## Discussion

### Method development and optimization

Proteins in cetacean blubber tissue were extracted, quantified and identified. With all three extraction methods trialled here, there was variability in the total protein yields between individuals, between duplicate subsamples of the same individual and also between assays of the same extract. The variability may be the result of a combination of possible factors caused by the samples themselves including, for example, high levels of individual variation between porpoises, or fine-scale variation and patchiness of proteins within the tissue. The amount of total protein extracted could also vary depending on the physical properties of the tissue such that the effectiveness of the extraction may differ between samples with higher or lower lipid or collagen content, for example.

Other sources of variation are likely introduced during the different stages of the extraction protocol. Sources of variability in the extraction Methods 1 and 2 include homogenization, centrifugation, pipetting, evaporation and resuspension phases, which together lead to differences in the total amount of extracted or lost protein through the whole process. Specifically, it is likely that resuspension of the protein in solution was a problem for reliable and consistent measurement of the precipitated proteins using Methods 1 and 2. These losses as a result of the multi-stage processing procedures, together with the compounding errors associated with high inter- and intra-assay CVs mean that these are not reliable methods for total protein extraction.

A protein precipitation method would be favoured in order to have the possibility of re-suspending the protein content of the blubber in a chosen buffer or solution that is compatible with subsequent analytical techniques (total protein quantification, SDS-PAGE, ELISAs, proteome profiler arrays for example). However, processing Method 3, a simpler method with no precipitation and resuspension of the protein, and few processing steps, resulted in the most consistent results with the highest total protein yield. This processing method results in an extract that is more limited in terms of its potential downstream applications as RIPA buffer is not compatible with a number of assays for protein quantification. Nevertheless, it is the preferred extraction method for these preliminary proteomic investigations.

The causes of the extraction variation using Method 3 (~20%) are still to be determined. It is likely that the protein content varies at a fine scale through the blubber tissue which gives rise to the extraction variability seen here. This natural variability is therefore a challenge to the usefulness of this technique in determining precise quantities as oppose to gross differences. The applicability of the method will be dictated by the acceptable level of precision for a particular study. Further work to investigate the sources of this variability should be prioritized. Similarly, further work should address the best means of standardizing the amount of protein quantified from blubber extracts, instead of using wet weight of the tissue, as this may reduce some of the extraction variability.

1D SDS-PAGE was used here to separate and visualize, for the first time, the protein components of cetacean blubber tissue. Both the best band resolution and the highest number of bands were seen in the RIPA buffer extracts. This confirmed that this method increases both the total amount of protein extracted, as well as the range of proteins within the sample compared to Methods 1 and 2. Linking a ‘shotgun’ proteomics approach to 1D SDS-PAGE was used here to identify proteins and, thus, investigate both the types of proteins and the most abundant proteins present in blubber. This work is based on the notion that known proteins from other organisms can help to identify cetacean proteins by homology. While there may be subtle differences in both protein structure and function, cetacean myoglobin compared to myoglobin from terrestrial species for example (Holm *et al.*, 2016), it was assumed that the proteins within the tissue could be positively identified based on regions of sequence identity with known proteins from vertebrate species within the NCBI database. However, due to the adaptation of marine mammals to an aquatic environment, the potential for protein structural modification could be high for some proteins. This could therefore lead to sources of error during protein identification, and should be considered when aiming to identify molecules that could serve as indicators of physiological condition.

### Proteins identified

As the gel protein bands extracted were all of different molecular weights, from all sex and age classes that had suffered various causes of death, we captured a wide variety of proteins present in the tissue. The largest functional group were those involved in general cell function and metabolism. Within this group, the proteins were further classed into more specific functional roles including proteins involved in biosynthesis, antioxidant proteins, regulators of the cell cycle and signal transduction pathway proteins, to name just a few. The variety of the 163 proteins in this group with a range of different metabolic functions is in keeping with a recent transcriptomic study of Northern elephant seal (*Mirounga angustirostris*) blubber tissue which showed that the most significantly enriched pathway in the blubber transcriptome, compared to the human proteome, was metabolism (Khudyakov *et al.*, 2017). The identification of important metabolic factors could therefore provide insight into localized tissue function. For example, heat shock proteins were identified here in the blubber extracts, and changes in gene expression for the heat shock response were detected in the transcriptome of the elephant seals (Khudyakov *et al.*, 2017). Heat shock proteins are key cellular defences against stress and play crucial roles in the folding and unfolding of proteins, the transport and sorting of proteins, as well as cell-cycle control and signalling (Li and Srivastava, 2004). In phocid seals, greater requirements for heat shock proteins and other antioxidants have been hypothesized at certain times during the life cycle as a result of rapid protein synthesis and high metabolic fuel availability (Bennett *et al.*, 2014). Thus, expression of these proteins could provide insight into cellular and physiological stresses of individuals.

The second largest functional group were proteins involved in the immune response and inflammation. Some of the immune, and acute-phase response proteins that were identified included haptoglobulin, transferrin and four members of the complement pathway. This is in-keeping with the recognition of the extensive and direct involvement of white adipocytes in inflammation and the acute-phase response in other mammals (Frühbeck *et al.*, 2001). It has been shown that adipocytes synthesize all of the proteins involved in the alternative complement pathway, specifically, factor C3. However, further research is required to determine the primary functions and regulation of this pathway in adipose tissue (Frühbeck *et al.*, 2001). Other proteins involved in immune system function were also identified including immunoglobulins (IgA and IgG) which, together, were the second most abundant proteins in the extracts. These were likely either in the circulation or were secreted directly from B-cell infiltrates within the blubber itself. Annexins, cyclophilins and dermicidin were among the other proteins identified that are involved in the regulation of inflammation and the immune response. Both innate and the adaptive components of the immune system were therefore present in the tissue. Given the current understanding of the involvement of adipose tissue in immune system function, it is possible that cetacean blubber could show a similar role, and the presence of such proteins could provide information regarding immune system function and activation in these animals.

As expected, there were also a range of factors present that play key roles in lipid metabolism, and these made up the third largest functional group of identified proteins. Fatty acid-binding proteins are low molecular-weight cytoplasmic proteins, and were particularly abundant here. The adipose-specific fatty acid-binding protein has been shown to be involved in intracellular trafficking and targeting of fatty acids (Frühbeck *et al.*, 2001), and may modulate lipolytic rate. Enzymes involved in lipolysis, as well as regulatory proteins were identified, including the hormone adiponectin. Adiponectin is an adipokine produced by white adipose tissue and released into the circulation, and is important for whole body metabolic regulation by increasing adipogenesis and lipid storage in fat tissue, as well as increasing insulin sensitivity (Fu *et al.*, 2005). Circulating adiponectin concentrations have been negatively correlated with total body fat stores in a number of terrestrial mammals (for a review see Fain *et al.* 2004), and, in marine mammals specifically, adiponectin is thought to be important in the development of blubber reserves in grey seal (*Halichoerus grypus*) pups (Bennett *et al.*, 2015). Concentrations of this hormone in the blubber could therefore provide information on the physiological state of an individual in terms of current energy stores. Adiponectin signalling pathways were also identified in the transcriptomic study of Northern elephant seals when investigating the acute metabolic response to glucocorticoids (Khudyakov *et al.*, 2017). Other differential gene expression was measured that promoted lipid catabolism and oxidation at the expense of lipid synthesis and storage (Khudyakov *et al.*, 2017). The presence of factors involved in various stages of lipid metabolism could be used to assess whether the individual is currently undergoing a period of lipolysis or lipogenesis. This is a good example of how the integration of proteomic and transcriptomic methods could result in a powerful assessment tool. Moving forward, the quantification of individual proteins could be coupled to the transcriptome so that transcription and translation can be linked.

Finally, although every attempt was made to obtain very fresh tissue samples, they were nevertheless collected from dead-stranded animals, so minor autolysis could have affected our findings. This would also complicate the functional interpretation of any proteins involved in *ante-* or *post-mortem* metabolic processes. However, the wide range of proteins and peptides identified here across a variety of metabolic pathways and processes suggests that proteomics is a robust tool to investigate tissue function using this approach.

### Tissue specific and circulatory proteins

While a range of different molecular weight proteins were identified, there may be some size selective loss of protein species as well as some loss of the least abundant proteins through the extraction procedure. There may also be some loss of more hydrophobic proteins that are more difficult to solubilize. Thus, if a protein was not identified following nLC-ESI MS/MS, this does not confirm its absence from the tissue, but this work does suggest that there are some proteins, and protein classes that are more abundant than others. Serum albumin and haemoglobin, from the circulation, were two of the most abundant proteins identified, and likely affected the detection of other proteins by swamping the samples. Further efforts to remove the albumin from the extracts would likely be required to detect less abundant proteins of potential interest. This could be achieved by fractionation of the extract for example or, the use of ‘Cibacron Blue’, a commercially available resin to remove albumins from solution. Alternatively, antibody columns could be used to target specific proteins. Similarly, targeted mass spectrometry could be used to detect particular peptides from the protein of interest to therefore detect the presence of proteins at lower concentrations within the samples.

Proteins identified here probably do not originate from solely the blubber tissue itself, but are a mixture of blubber proteins together with plasma proteins. Attempts were made to limit any external blood on the samples by using visibly ‘cleaner’ parts of the tissue. The presence of plasma proteins in the extracts were likely largely a result of their presence *within* the tissue vasculature rather than *on* each piece of tissue from contaminating sources during the necropsy sampling. The vascularization of marine mammal blubber tissue is still not well understood, but one study comparing the microvasculature of deep diving and shallow diving odontocetes saw that blubber tissue is more highly vascularised than adipose tissue in terrestrial mammals (McClelland *et al.*, 2012). As such, blubber tissue sampling can provide information on both the proteins produced and metabolized *in situ* as well as those in circulation. Double sampling of freshly dead stranded animals before blood coagulation occurs would be useful here for further comparisons of proteins present in both the blood and blubber of the same individuals. This would be an important next step to identify those proteins present in both matrices, and those that are found more exclusively in the blubber itself.

The huge range of proteins identified here clearly demonstrate the heterogeneity of blubber tissue, and a comparison of the proteins present in the inner blubber layers and the outer layers would help to establish if this heterogeneity results from longitudinal differences in metabolic activity and/or different cell types. While it has been well established that adipocytes express and secrete several endocrine hormones, many secreted proteins within the tissue are derived from the non-adipocyte fraction (Fain *et al.*, 2004). For example, the innermost blubber layer has been shown to consist of a heterogeneous mix of white adipocytes, brown adipocytes and connective tissue as well as muscle and nerve fibres (Hashimoto *et al.* 2015). Therefore, as the full depth blubber layer was sampled, proteins detected here were the result of differential gene expression in these different cell and tissue types which explains the presence of myoglobin across all samples. Thus, as well as the origin of certain proteins in terms of either the blubber or the circulatory system, an important next step would also be to establish the secreting cell types within the tissue. It would be possible to determine whether gene expression and protein secretion occurs within the mature adipocytes, or in the other cells that make up the tissue, either histologically (through *in situ* hybridization) or by separation of the adipocytes from the stromal vascular fraction by collagenase digestion (Trayhurn and Wood, 2004).

### Potential for biomarker development

There is a need to develop appropriate markers of nutritive condition, health and physiological state in free-ranging cetaceans where the ‘standard’ set of biological samples (blood, faeces and urine) and morphometric measurements cannot be routinely obtained from live animals. Blubber and skin biopsy samples, however, can be obtained through remote sampling (Hunt *et al.*, 2013). To date, in order to estimate an individual’s condition, analysis of biopsy samples has focused on the total lipid content as a marker of nutritive status (Krahn *et al.*, 2004). However, this approach has problems associated with the loss of lipid from the sample upon collection (Ryan *et al.*, 2012), and the stratification of lipid through the blubber depth (Koopman *et al.*, 1996; Olsen and Grahl-Nielsen, 2003; Smith and Worthy, 2006). Another potential approach to infer condition is to measure the size of the adipocytes themselves which may be more promising (Castrillon *et al.*, 2017).

Other studies have established the reproductive status of individuals through the measurement of concentrations of reproductive hormones in blubber, specifically progesterone and testosterone (Kellar *et al.*, 2009, 2013). The concentrations of blubber cortisol have been measured in terms of both an indicator of physiological state and body condition (Kershaw *et al.*, 2017) as well as the stress response (Kellar *et al.*, 2015; Champagne *et al.*, 2017). Moving forward, the identification and quantification of different hormones and proteins involved in various metabolic pathways within blubber tissue could lead to the development of potential new protein markers of interest that can be quantified to provide information on a range of physiological processes and life history states. Explaining and quantifying the natural variability in these protein markers in the context of different life histories or causes of death is the next step in developing this approach.

Indicator proteins may be produced directly by the blubber itself, secreted either by the adipocytes, the stromovascular cells, or a combination of both, or may have accumulated in the tissue from the circulation in a manner dependent on the individual’s metabolic state. They therefore have the potential to capture information on a range of different metabolic processes and provide insight into the physiological stresses experienced by individuals. Of particular interest to assess energy stores and body condition would be the adipokines such as adiponectin, detected here, as well was various protein factors involved in lipid metabolism. In addition, the range of immune proteins identified suggests that the blubber could be a valuable tissue for assessing immune system function and inflammatory responses. Overall, proteomic studies have the potential to identify key metabolic processes and pathways and therefore assign novel functional roles to marine mammal blubber tissue.

## Supplementary Material

Supplementary Data
