# Supplementary material for: Not just fat: investigating the proteome of cetacean blubber tissue
Source: Conserv Physiol. 2018 Feb 16;6(1):coy003. doi: 10.1093/conphys/coy003 (PMC5814904; doi:10.1093/conphys/coy003)
Supplement: Supplementary Data [file coy003revisedsupplementarymaterials.kershawetal.cetaceanblubberproteome.pdf]

SUPPLEMENTARY MATERIALS

Not just fat: Investigating the proteome of cetacean blubber tissue

***Joanna L. Kershaw[1], Catherine H. Botting[2], Andrew Brownlow[3], Ailsa J. Hall[1]**

*Corresponding author: jk49@st-andrews.ac.uk

[1] Sea Mammal Research Unit, Scottish Oceans Institute, University of St Andrews, St Andrews, KY16 8LB, UK

[2] Biomedical Sciences Research Complex, School of Chemistry, Biomolecular Sciences Building Annexe, North Haugh, University of St Andrews, St Andrews, KY16 9ST, UK

[3] Scottish Marine Animals Strandings Scheme, SAC Consulting Veterinary Services, Inverness, IV2 5NA, UK

SUPPLEMENTARY METHODS

1. Total Protein Extraction Methods

1.1 Methanol-Chloroform Precipitation with Methanol Pellet Wash

16 full depth blubber subsamples were accurately weighed (0.4 - 0.6g) and homogenised with 0.5mL of isolation medium (50mM Tris, 150mM NaCl, 0.2mM EDTA and 10ug/ml protease inhibitors), and 1.875mL of 1:2 chloroform/methanol. Samples were then placed on ice for 15 minutes with thorough mixing every three minutes. 625ul of chloroform and 625ul of deionised water were added to the sample. The sample was vortexed and centrifuged at 800g for 5 minutes at 4^0C. This creates three layers, a lower lipid layer, an upper protein layer and a protein disc between the two. The upper protein phase and the protein disc were collected and transferred to a new tube.

Three volumes of water, four volumes of methanol and one volume of chloroform were added to the solution and vortexed vigorously for 5 min so that only one phase was visible in the tube. The mixture was centrifuged at 4700g at 4^0C for 30 min. This forms two immiscible layers with the protein precipitate at the interface between the two. The water /methanol top layer was removed, and care was taken not to disturb the interface as often precipitated proteins do not form a visibly white surface. Another four times the volume of methanol were added to wash the precipitate, and the mixture was again vortexed vigorously for 5 min. The mixture was centrifuged at 4700g at 4^0C for 45 min. This forms a protein pellet in the bottom of the tube. The supernatant was removed and the pellet was dried under N_2 at room temperature. Care was taken not to over dry the pellet causing it to become flaky and stick to the centrifuge tube which reduces the resuspension efficiency. Finally, the pellet was resuspended in 500ul of SDS/Tris (0.1% SDS in 40mM Tris).

1.2 TCA-Acetone Precipitation with Acetone Pellet Wash

Using another 16, different, full-depth blubber subsamples, the same protocol as previously described was followed up to the collection of the upper protein phase and the protein disc, and its transfer to a new tube. 10% TCA in acetone solution was added to the sample with a ratio of 1:3 of sample to TCA-acetone solution. The sample was frozen overnight at -20^0C. The sample was allowed to warm to room temperature and then centrifuged at 4700g at 4^0C for 45 minutes the following day. Proteins form a pellet in the bottom of the tube. The pellet was washed twice by adding 1ml of ice cold acetone which was then discarded. Any remaining acetone following the final wash was evaporated under nitrogen at room temperature. Care was taken not to over-dry the protein pellet, and the pellet was resuspended in 500ul of SDS/Tris. Of the two protein precipitation methods, using a TCA-acetone solution showed least total protein inter-assay variability. For this reason, alterations were made to improve this protocol in an attempt to

firstly, further clean the extract, and secondly, to improve protein pellet resuspension by altering the pellet washing procedure.

Alteration 1: TCA-Acetone Precipitation with Butanol De-Lipidation and Acetone Pellet Wash

As cetacean blubber tissue has such a high lipid content, an extra butanol de-lipidation step was added before the precipitation of the protein pellet in an attempt to further 'clean' the extract (Zhao and Xu, 2010). Another 16 blubber subsamples were taken, as previously described, from the same individuals used for the TCA-acetone precipitation protocol. Following the collection of the upper protein phase and protein disc (after homogenisation and centrifugation), butanol was added in a ratio of 1:3 sample to butanol. This was then centrifuged at 1750g at 10^0C for 10 min and the upper phase containing any remaining lipids was removed and discarded. 10% TCA in acetone was added to the lower phase, and the precipitation and pellet wash continued as described above. The final protein pellet was resuspended in 500ul of SDS/Tris.

Alteration 2: TCA-Acetone Precipitation with Water Wash

Poor resuspension of the protein pellet in the SDS-Tris Buffer could result in under-estimates of total protein content if some pellets resuspend less efficiently than others. The effect of a different pellet wash was tested by using de-ionised water instead of acetone for the final wash stage. This was an attempt not to dehydrate the pellet, and therefore make its resuspension back into solution more efficient. As before, 16 blubber subsamples were taken from the same eight individuals used for the TCA-acetone precipitation protocol. Specifically, following the precipitation of the proteins in TCA-acetone, care was taken to discard all TCA-acetone solution, and the pellet was washed by adding 1ml of deionised water. It was vortexed briefly, and then centrifuged again for 5 min at 4700g at 4^0C. The supernatant was again discarded, and the pellet washed a second time with deionized water. Care was taken not to disturb the washed pellet that was then re-suspended in 500ul of SDS/Tris.

1.3 RIPA Lysis and Extraction Buffer

Another 16, full depth blubber samples were used for analysis from 8 individuals. The subsamples were accurately weighed (0.1 ± 0.01g). The frozen tissue was placed on ice in a 1.5ml low protein binding micro-centrifuge tube and 200ul RIPA lysis and extraction buffer (Thermo Fisher Scientific) with 2X concentration of protease inhibitors (Pierce Protease Inhibitor Mini Tablets) was added to the tissue. The samples were homogenised with a pestle designed for a 1.5ml tube for two minutes to give a cloudy white solution and small piece of connective tissue that could not be further homogenised. The samples were replaced on ice and then centrifuged at 4700g at 4^0C for 15 minutes before being replaced on ice. The supernatant, the hardened lipid containing fraction, was pushed to one side, and the protein containing infranatant was removed and placed into a clean, low protein binding microcentrifuge tube. Care

was taken not to disturb the insoluble/unhomogenised connective tissue pellet. The protein fraction was centrifuged, as before, as a second de-lipidation step, and replaced on ice. Again, the infranatant was removed and placed in a clean, low protein binding microcentrifuge tube taking care not to disturb any remaining lipid supernatant and insoluble pellet.

2. Total Protein Quantification Assays

To assess the effectiveness of the different protein extraction methods, two different commercially available total protein quantification kits were used to measure the protein in the blubber extracts. Firstly, a Pierce™ BCA Protein Assay Kit (23225, Thermo Scientific, Rockford, USA) was used to quantify the total protein in the extracts resuspended in SDS-Tris using the chloroform-methanol and TCA-acetone precipitation methods. The assay is a detergent-compatible formulation based on bicinchoninic acid (BCA) for the colorimetric detection and quantitation of total protein. This assay has a broad working range of protein concentrations between 20-2000µg/mL. A series of dilutions of known concentrations of bovine serum albumin were prepared using the same SDS/Tris used to re-suspend the protein extracts, and assayed alongside blubber extracts to determine their protein concentrations based on the standard curve produced using a log-logistic 4 parameter model. As blubber extract sample volumes are small, the microplate procedure was chosen for this analysis and assays were carried out following the kit instructions. Each extract was assayed in duplicate on every plate, a negative control of SDS/Tris was run on every plate, and total protein concentrations were reported as µg per wet weight of the sample. Each extract was assayed on three different plates in order to determine the inter-assay coefficient of variation (% CV), and the average concentration measured across the three plates was used as the final estimated total protein content of a sample.

Secondly, a Pierce™ 660nm Protein Assay (22662, Thermo Scientific, Rockford, USA) was used to quantify the total protein in the extracts processed in RIPA lysis and extraction buffer. This is a quick, ready-to-use colorimetric method for total protein quantitation, and is compatible with high concentrations of most detergents and reducing agents, and is therefore compatible with the RIPA buffer supplemented with 2X protease inhibitors used here. The blubber extracts were prepared following the assay instructions for cell lysates in RIPA buffer by adding Triton™ X-100 to a final concentration of 0.8% to the sample before performing the assay. A series of dilutions of known concentrations of bovine serum albumin were used to generate a standard curve. A negative control of RIPA with 2X protease inhibitors was used as the zero standard on each plate, and the absorbance measured was used to correct the absorbance of the standard curve measurements which were then used to produce a 4 parameter log-logistic model and calculate the protein concentrations in the samples. The assay was performed following the microplate protocol and the absorbance read at 660nm. Each extract was assayed in duplicate and total protein concentrations are reported as µg per wet weight of the sample. Each extract was assayed on two different plates in order to determine the inter-assay CV (% CV), and the

average concentration measured across the two plates was used as the final estimated total protein content of a sample.

3. 1D SDS-PAGE Protein Separation

The final stage of the method development was to optimise protein separation and visualisation using 1D SDS-PAGE (Sodium Dodecyl Sulfate - Polyacrylamide Gel Electrophoresis) which separates denatured proteins based on their molecular weight. The XCell SureLock® Mini-Cell (Life Technologies, UK) gel electrophoresis unit was used to run 4-12% NuPAGE Bis-Tris mini gels (8.0cm x 8.0cm x 1.0mm) (NP0321BOX, Thermo Fisher Scientific, Paisley, UK) with a wide protein separation range of 3.5 kDa to 160 kDa. The recommended sample and running buffers for de-natured proteins as listed by the manufacturers were used to run the gels: 1x NuPAGE LDS Sample Buffer (NP0007, Thermo Fisher Scientific, Paisley, UK) and 1x NuPAGE MES SDS Running Buffer (NP000202, Thermo Fisher Scientific, Paisley, UK). Standard running conditions as recommended by the manufacturers were also chosen: a constant voltage of 200V and a current of 125mA for 35 minutes. A wide protein range ladder, Invitrogen™ Novex™ Mark 12™ Unstained Standard (LC5677, Fisher Scientific, USA) was run on each gel. The blubber extracts resuspended in SDS-Tris were run undiluted on the gels while the extracts in RIPA buffer were run at ½ and $^{1}/_{3}$ dilutions as the protein content of these extracts was much higher. 5µl of NuPAGE LDS Sample Buffer (4x) was added to 20µl of each of the blubber extracts (neat or diluted). 10µl of each of these were loaded onto the gel. Upon completion of the run, the gels were removed from the cassettes and rinsed in de-ionised water briefly to remove residual SDS that affects the staining procedure. The gels were stained in 500ml of Bio-Safe Coomassie Stain (1610786, Bio-Rad, UK) for 1.5 hours with gentle agitation on a platform shaker. This stain is fast, simple, sensitive and convenient in it's ready to use and non-hazardous solution. The gels were then removed from the stain, rinsed and detained in de-ionised water overnight. The de-stained gels were photographed on a white background using a BioDoc-It™ Imaging System (Ultra-Violet Products Ltd, Cambridge, UK).

SUPPLEMENTARY RESULTS

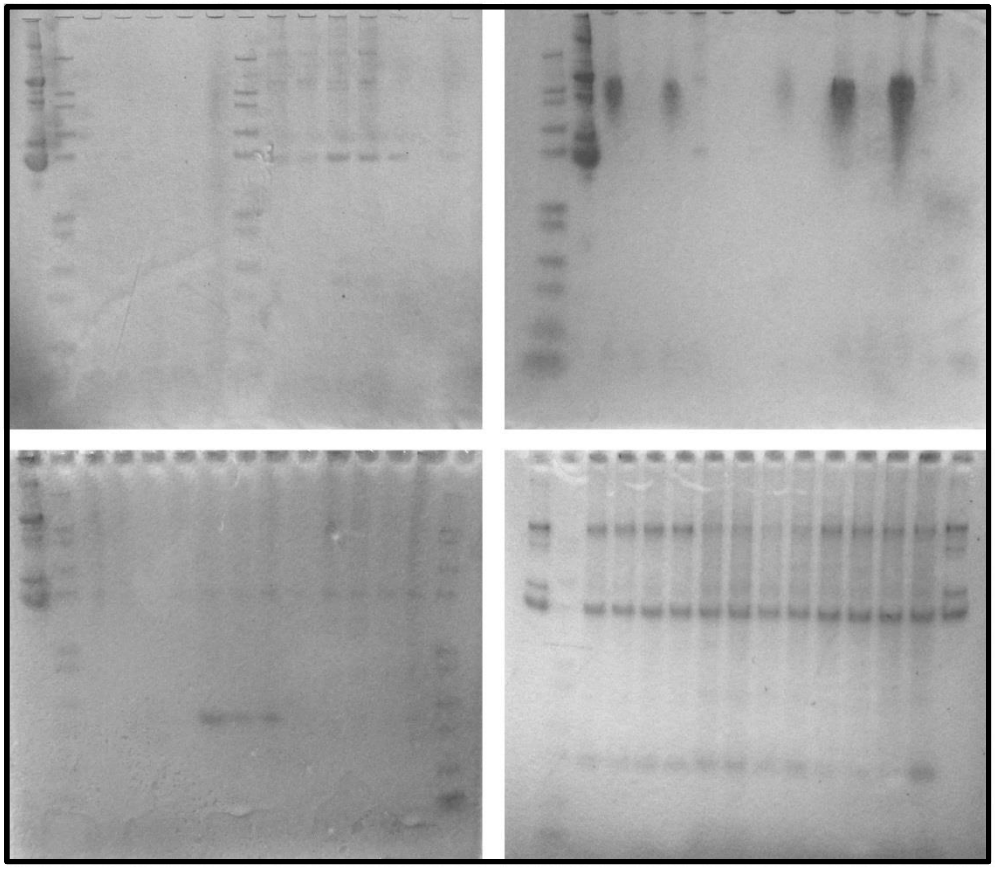

Fig. 1. Example images of 1D SDS-PAGE analysis of harbour porpoise blubber tissue extracts processed using Methods 1 and 2 on 4-12% Bis-Tris gels stained with Bio-Safe Coomassie brilliant blue. Extracts show few protein bands only across the larger size range. There is high background and some protein smearing. In addition, there is poor reproducibility of bands across extracts and across gels.

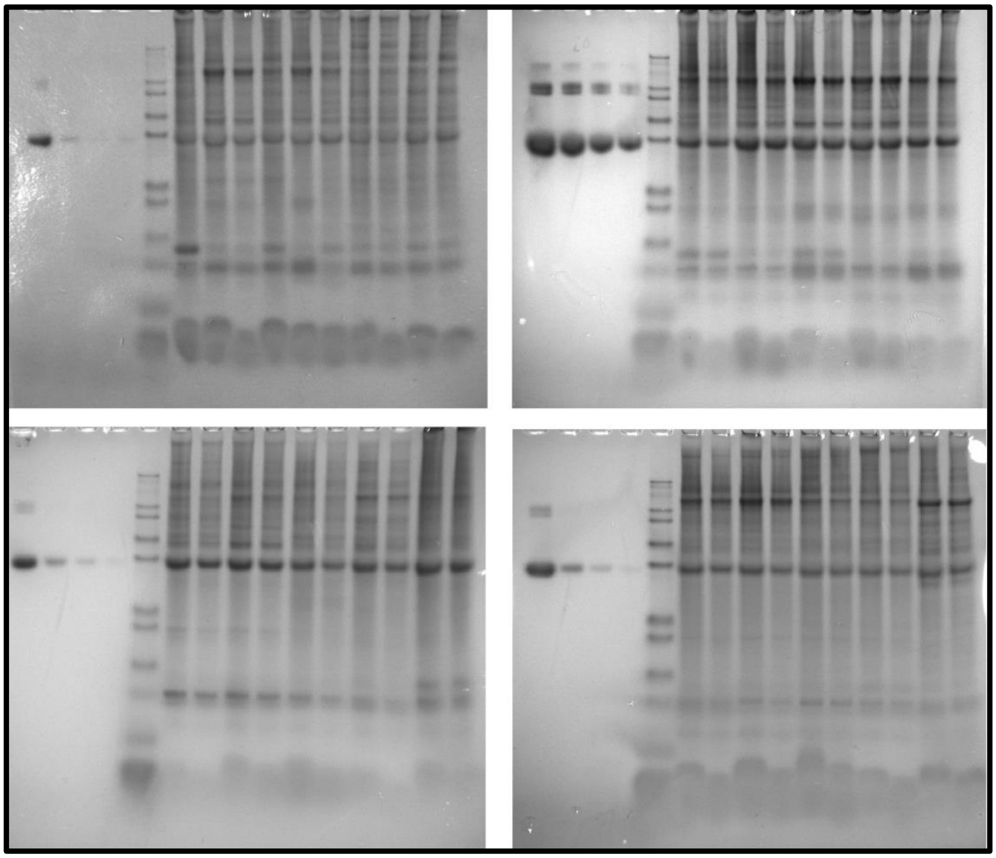

Fig. 2. Example images of 1D SDS-PAGE analysis of harbour porpoise blubber tissue extracts processed using Methods 3 on 4-12% Bis-Tris gels stained with Bio-Safe Coomassie brilliant blue. Extracts show a large number of protein bands over a wide range of protein sizes. Protein taining is high with minimal background and smearing. Reproducibility of bands across extracts and across gels is excellent.

Table 1. 295 proteins, with 2 or more peptides with Mascot Ion Scores above the Identity ($p<0.05$) Threshold, identified in the harbour porpoise blubber extracts grouped by functional group. The total frequency is the number of times the protein was identified across all 36 samples analysed by nLC-ESI-MS/MS.

Protein Name	Protein Type	Protein Group	Specific Function	Total Frequency
4-trimethylaminobutyraldehyde dehydrogenase	Enzyme	Amino Acid Metabolism		1
dipeptidase	Enzyme	Amino Acid Metabolism		1
leucine aminopeptidase	Enzyme	Amino Acid Metabolism		1
trypsin inhibitor	Enzyme	Amino Acid Metabolism		1
xaa-Pro aminopeptidase 1	Enzyme	Amino Acid Metabolism		1
Xaa-Pro dipeptidase	Enzyme	Amino Acid Metabolism		1
translation factor GUF1 homolog	Regulatory	Amino Acid Metabolism		1
3-hydroxy-2-methylbutyryl-CoA dehydrogenase	Enzyme	Amino Acid Metabolism		1
insulin-activated amino acid transporter	Transport	Amino Acid Metabolism		2
aminotransferase	Enzyme	Amino Acid Metabolism		3
cyclase-associated protein	Regulatory	Cell Function and Metabolism	Actin-Binding	1
macrophage-capping protein	Regulatory	Cell Function and Metabolism	Actin-Binding	1
AHNAK nucleoprotein	Regulatory	Cell Function and Metabolism	Adapter	1
cellular retinoic acid-binding protein 3	Regulatory	Cell Function and Metabolism	Adapter	1
cytochrome b5	Transport	Cell Function and Metabolism	Adapter	1
MICOS complex subunit MIC61	Regulatory	Cell Function and Metabolism	Adapter	1
mitochondrial fission 1 protein	Regulatory	Cell Function and Metabolism	Adapter	1

plasma membrane calcium ATPase	Regulatory	Cell Function and Metabolism	Adapter	1
prostaglandin F2 receptor negative regulator	Regulatory	Cell Function and Metabolism	Adapter	1
puromycin-sensitive aminopeptidase	Enzyme	Cell Function and Metabolism	Adapter	1
RNA-binding motif protein 3	Regulatory	Cell Function and Metabolism	Adapter	1
serpin peptidase inhibitor	Regulatory	Cell Function and Metabolism	Adapter	1
SH3 domain-binding glutamic acid-rich-like protein	Regulatory	Cell Function and Metabolism	Adapter	1
solute carrier family proteins	Regulatory	Cell Function and Metabolism	Adapter	1
SSR	Regulatory	Cell Function and Metabolism	Adapter	1
succinate dehydrogenase flavoprotein	Enzyme	Cell Function and Metabolism	Adapter	1
synaptic vesicle membrane protein VAT-1	Regulatory	Cell Function and Metabolism	Adapter	1
TPA: heparan sulfate proteoglycan 2	Regulatory	Cell Function and Metabolism	Adapter	1
peptidyl-prolyl cis-trans isomerase B	Enzyme	Cell Function and Metabolism	Adapter	2
transitional endoplasmic reticulum ATPase	Enzyme	Cell Function and Metabolism	Adapter	2
14-3-3 protein	Regulatory	Cell Function and Metabolism	Adapter	3
gelsolin	Regulatory	Cell Function and Metabolism	Adapter	4
phosphate carrier protein	Transport	Cell Function and Metabolism	Adapter	4
platelet glycoprotein 4-like	Regulatory	Cell Function and Metabolism	Adapter	7
periostin	Regulatory	Cell Function and Metabolism	Adapter	9
biglycan	Regulatory	Cell Function and Metabolism	Adapter	10
adenine phosphoribosyltransferase	Enzyme	Cell Function and Metabolism	Biosynthesis	1
glycyl-tRNA synthetase	Enzyme	Cell Function and Metabolism	Biosynthesis	1

iron response element binding protein	Regulatory	Cell Function and Metabolism	Biosynthesis	1
nucleoside diphosphate kinase A	Enzyme	Cell Function and Metabolism	Biosynthesis	1
asparagine synthetase	Enzyme	Cell Function and Metabolism	Biosynthesis	2
enhancer protein	Regulatory	Cell Function and Metabolism	Biosynthesis	2
glutamate dehydrogenase (NAD(P)+)	Enzyme	Cell Function and Metabolism	Biosynthesis	2
protein glycosyltransferase	Enzyme	Cell Function and Metabolism	Biosynthesis	2
elongation factors	Regulatory	Cell Function and Metabolism	Biosynthesis	4
S100A4	Regulatory	Cell Function and Metabolism	Cell Adhesion	1
transforming growth factor-beta-induced protein ig-h3	Regulatory	Cell Function and Metabolism	Cell Adhesion	3
cell surface glycoprotein MUC18	Regulatory	Cell Function and Metabolism	Cell Adhesion	5
homeodomain-interacting protein kinase 2-like	Enzyme	Cell Function and Metabolism	Cell Cycle	1
olfactomedin-like 1	Regulatory	Cell Function and Metabolism	Cell Cycle	1
PEA-15	Regulatory	Cell Function and Metabolism	Cell Cycle	1
T-cell leukemia homeobox protein 3	Regulatory	Cell Function and Metabolism	Cell Cycle	1
carnitine O-acetyltransferase	Enzyme	Cell Function and Metabolism	Cell Cycle	4
ADP/ATP translocase 3	Regulatory	Cell Function and Metabolism	Channel	1
insulin-like growth factor II receptor	Regulatory	Cell Function and Metabolism	Channel	1
large neutral amino acids transporter	Transport	Cell Function and Metabolism	Channel	1
LETM1 and EF-hand domain-containing protein 2	Regulatory	Cell Function and Metabolism	Channel	1
mannose-6-phosphate receptor	Regulatory	Cell Function and Metabolism	Channel	1
vacuolar H+-ATPase	Enzyme	Cell Function and Metabolism	Channel	1

ATP synthase	Enzyme	Cell Function and Metabolism	Channel	5
4F2 antigen	Regulatory	Cell Function and Metabolism	Channel	5
endoplasmin	Regulatory	Cell Function and Metabolism	Chaperone	1
hsc70-interacting protein	Regulatory	Cell Function and Metabolism	Chaperone	1
importin	Transport	Cell Function and Metabolism	Chaperone	1
lanC-like protein 1	Regulatory	Cell Function and Metabolism	Chaperone	1
protein deglycase DJ-1	Regulatory	Cell Function and Metabolism	Chaperone	1
T-complex protein 1	Regulatory	Cell Function and Metabolism	Chaperone	1
calreticulin	Regulatory	Cell Function and Metabolism	Chaperone	2
calnexin	Regulatory	Cell Function and Metabolism	Chaperone	3
protein disulfide-isomerases	Enzyme	Cell Function and Metabolism	Chaperone	5
DNA polymerase	Enzyme	Cell Function and Metabolism	DNA Metabolism	1
histones	Regulatory	Cell Function and Metabolism	DNA Metabolism	3
ADP-ribosylation factor 2-like	Regulatory	Cell Function and Metabolism	GTPase	1
rab GDP dissociation inhibitor	Regulatory	Cell Function and Metabolism	GTPase	1
Rab5c-like protein	Regulatory	Cell Function and Metabolism	GTPase	1
rho GDP-dissociation inhibitor 2	Regulatory	Cell Function and Metabolism	GTPase	1
heat shock protein 84	Regulatory	Cell Function and Metabolism	Heat Shock Protein	1
heat shock protein 60	Regulatory	Cell Function and Metabolism	Heat Shock Protein	2
heat shock protein 90	Regulatory	Cell Function and Metabolism	Heat Shock Protein	2
heat shock protein 70	Regulatory	Cell Function and Metabolism	Heat Shock Protein	4

S-adenosylhomocysteine hydrolase	Enzyme	Cell Function and Metabolism	Hydrolase	1
proteasome subunits	Regulatory	Cell Function and Metabolism	Hydrolase	8
2-oxoglutarate dehydrogenase	Enzyme	Cell Function and Metabolism	Metabolism	1
6-phosphofructokinase	Enzyme	Cell Function and Metabolism	Metabolism	1
Adenylate monophosphate kinase	Enzyme	Cell Function and Metabolism	Metabolism	1
aspartyl beta-hydroxylase	Enzyme	Cell Function and Metabolism	Metabolism	1
Atp5b protein	Enzyme	Cell Function and Metabolism	Metabolism	1
delta-1-pyrroline-5-carboxylate dehydrogenase	Enzyme	Cell Function and Metabolism	Metabolism	1
flavin reductase (NADPH)	Enzyme	Cell Function and Metabolism	Metabolism	1
Methylmalonyl-CoA decarboxylase	Enzyme	Cell Function and Metabolism	Metabolism	1
NADP-dependent malic enzyme	Enzyme	Cell Function and Metabolism	Metabolism	1
phosphoglycerate mutase 2	Enzyme	Cell Function and Metabolism	Metabolism	1
prohibitin	Regulatory	Cell Function and Metabolism	Metabolism	1
retinal dehydrogenase 1	Enzyme	Cell Function and Metabolism	Metabolism	1
Rieske Fe-S protein	Regulatory	Cell Function and Metabolism	Metabolism	1
glucose-6-phosphate dehydrogenase	Enzyme	Cell Function and Metabolism	Metabolism	2
PKM2 protein	Enzyme	Cell Function and Metabolism	Metabolism	2
6-phosphogluconate dehydrogenase	Enzyme	Cell Function and Metabolism	Metabolism	2
aconitate hydratase	Enzyme	Cell Function and Metabolism	Metabolism	2
glutathione S-transferase P-like	Enzyme	Cell Function and Metabolism	Metabolism	2
pyruvate dehydrogenase	Enzyme	Cell Function and Metabolism	Metabolism	2

sodium/potassium-transporting ATPase	Enzyme	Cell Function and Metabolism	Metabolism	2
transaldolase	Enzyme	Cell Function and Metabolism	Metabolism	2
creatine kinases	Enzyme	Cell Function and Metabolism	Metabolism	3
cytochrome b5 reductase	Enzyme	Cell Function and Metabolism	Metabolism	3
transketolase	Enzyme	Cell Function and Metabolism	Metabolism	5
aldolases	Enzyme	Cell Function and Metabolism	Metabolism	5
triosephosphate isomerase	Enzyme	Cell Function and Metabolism	Metabolism	7
pyruvate kinase	Enzyme	Cell Function and Metabolism	Metabolism	7
GAPDH	Enzyme	Cell Function and Metabolism	Metabolism	9
amine oxidase	Enzyme	Cell Function and Metabolism	Metabolism	10
carbonic anhydrases	Enzyme	Cell Function and Metabolism	Metabolism	10
lactate dehydrogenase	Enzyme	Cell Function and Metabolism	Metabolism	13
ethylmalonyl-CoA decarboxylase	Enzyme	Cell Function and Metabolism	Protective	1
selenium-binding protein 1	Regulatory	Cell Function and Metabolism	Protective	1
transmembrane protein 109	Regulatory	Cell Function and Metabolism	Protective	2
aspartyl aminopeptidase	Enzyme	Cell Function and Metabolism	Protein Dedgadation / Modification	1
GANAB protein	Enzyme	Cell Function and Metabolism	Protein Dedgadation / Modification	1
ubiquitin-like modifier-activating enzyme 1	Enzyme	Cell Function and Metabolism	Protein Dedgadation / Modification	1
cAMP-dependent protein kinase	Enzyme	Cell Function and Metabolism	Protein Dedgadation / Modification	2
CAND1 protein	Regulatory	Cell Function and Metabolism	Protein Degradation /Modifiation	1
dolichyl-diphosphooligosaccharide--protein glycosyltransferase	Enzyme	Cell Function and Metabolism	Protein Degradation / Modifiation	1

cathepsin D	Enzyme	Cell Function and Metabolism	Protein Degradation / Modifiation	2
phosphatidylethanolamine-binding protein 1	Regulatory	Cell Function and Metabolism	Protein Degradation / Modifiation	2
prolyl endopeptidase	Enzyme	Cell Function and Metabolism	Protein Degradation / Modification	1
protein kinase	Enzyme	Cell Function and Metabolism	Protein Degradation / Modification	1
Protein kinase C inhibitor	Regulatory	Cell Function and Metabolism	Protein Degradation / Modification	1
pseudouridine-5'-phosphatase	Enzyme	Cell Function and Metabolism	Protein Degradation / Modification	1
signal peptidase complex	Enzyme	Cell Function and Metabolism	Protein Degradation / Modification	1
prenylcysteine oxidase 1	Enzyme	Cell Function and Metabolism	Protein Degradation / Modification	2
ubiquitin	Regulatory	Cell Function and Metabolism	Protein Degradaton / Modification	1
fetuin-B	Enzyme	Cell Function and Metabolism	Protein Degradaton / Modification	5
dihydrolipoamide dehydrogenase	Enzyme	Cell Function and Metabolism	Redox Protein	1
manganese superoxide dismutase	Enzyme	Cell Function and Metabolism	Redox Protein	1
redox-regulatory protein FAM213A	Regulatory	Cell Function and Metabolism	Redox Protein	1
thioredoxin	Regulatory	Cell Function and Metabolism	Redox Protein	1
thioredoxin peroxidase	Enzyme	Cell Function and Metabolism	Redox Protein	1
thioredoxin reductase	Enzyme	Cell Function and Metabolism	Redox Protein	1
Cu/Zn superoxide dismutase	Enzyme	Cell Function and Metabolism	Redox Protein	2
glutathione peroxidase	Enzyme	Cell Function and Metabolism	Redox Protein	2
thioredoxin-dependent peroxide reductase	Enzyme	Cell Function and Metabolism	Redox Protein	2
microsomal glutathione S-transferase 1	Enzyme	Cell Function and Metabolism	Redox Protein	3
catalase	Enzyme	Cell Function and Metabolism	Redox Protein	4

peroxiredoxin	Enzyme	Cell Function and Metabolism	Redox Protein	4
polyadenylate-binding protein 1	Regulatory	Cell Function and Metabolism	RNA Metabolism	1
ribonucleoprotein	Regulatory	Cell Function and Metabolism	RNA Metabolism	1
RNA polymerase	Enzyme	Cell Function and Metabolism	RNA Metabolism	1
ribonuclease inhibitor	Regulatory	Cell Function and Metabolism	RNA Metabolism	2
nucleolin	Structural	Cell Function and Metabolism	RNA Metabolism	3
ribosomal proteins	Regulatory	Cell Function and Metabolism	RNA Metabolism	7
eukaryotic translation initiation factor 4	Regulatory	Cell Function and Metabolism	RNA Regulation	1
Gry-rbp	Regulatory	Cell Function and Metabolism	RNA Regulation	1
NF45 protein	Regulatory	Cell Function and Metabolism	RNA Regulation	1
DEAD box protein RB	Regulatory	Cell Function and Metabolism	RNA Regulation	2
heterogeneous nuclear ribonucleoprotein R	Regulatory	Cell Function and Metabolism	RNA Regulation	3
lupus La protein	Regulatory	Cell Function and Metabolism	RNA Regulation	3
dapper homolog 2	Regulatory	Cell Function and Metabolism	Signal Transduction	1
mitochondrial import receptor subunit TOM70	Regulatory	Cell Function and Metabolism	Signal Transduction	1
phospholipase c delta 1	Enzyme	Cell Function and Metabolism	Signal Transduction	1
phosphoprotein phosphatase	Enzyme	Cell Function and Metabolism	Signal Transduction	1
CD81 antigen	Regulatory	Cell Function and Metabolism	Signal Transduction	2
Transforming protein RhoA	Regulatory	Cell Function and Metabolism	Signal Transduction	2
polymerase I and transcript release factor	Regulatory	Cell Function and Metabolism	Signal Transduction	5
Ras proteins	Regulatory	Cell Function and Metabolism	Signal Transduction	6

ADP-ribosylation factor-like protein 8B	Regulatory	Cell Function and Metabolism	Trafficking	1
Alpha-soluble NSF attachment protein	Regulatory	Cell Function and Metabolism	Trafficking	1
clathrin	Regulatory	Cell Function and Metabolism	Trafficking	1
GTP-binding protein	Regulatory	Cell Function and Metabolism	Trafficking	1
huntingtin interacting protein	Regulatory	Cell Function and Metabolism	Trafficking	1
NIPSNAP2 protein	Regulatory	Cell Function and Metabolism	Trafficking	1
vesicle-trafficking protein SEC22b	Regulatory	Cell Function and Metabolism	Trafficking	1
caveolin	Regulatory	Cell Function and Metabolism	Trafficking	3
NADH dehydrogenase	Enzyme	Cell Function and Metabolism	Trafficking	4
profilin-1	Structural	Cell Structure	Actin Binding	1
protein 4.1	Structural	Cell Structure	Actin Binding	1
alpha-actinin-1	Structural	Cell Structure	Actin Binding	2
transgelin	Structural	Cell Structure	Actin Binding	2
F-actin-capping protein	Structural	Cell Structure	Actin-Binding	1
fascin	Structural	Cell Structure	Actin-Binding	1
cadherin-13	Structural	Cell Structure	Cell Adhesion	1
ezrin isoform X1	Structural	Cell Structure	Cell Adhesion	1
laminin-binding protein	Structural	Cell Structure	Cell Adhesion	1
microfibril-associated glycoprotein 4	Structural	Cell Structure	Cell Adhesion	1
mitogen inducible gene mig-2	Structural	Cell Structure	Cell Adhesion	2
integrins	Structural	Cell Structure	Cell Adhesion	4

fermitin homologue 2	Structural	Cell Structure	Cell Motility	1
WDR1 protein	Regulatory	Cell Structure	Cell Motility	1
moesin	Structural	Cell Structure	Cell Motility	2
p64 CLCP	Structural	Cell Structure	Cell Motility	2
vinculin	Structural	Cell Structure	Cell Motility	2
talin	Structural	Cell Structure	Cell Motility	4
stomatin peptide	Structural	Cell Structure	Membrane Protein	1
spectrin	Structural	Cell Structure	Membrane Protein	4
laminin	Structural	Cell Structure	Membrane Protein	11
T-plastin	Regulatory	Cell Structure	Structural Integrity	1
transmembrane protein 43	Structural	Cell Structure	Structural Integrity	1
vimentin	Structural	Cell Structure	Structural Integrity	9
adenylyl cyclase-associated protein	Structural	Cell Structure	Trafficking	1
coronin-3	Structural	Cell Structure	Trafficking	1
dynein	Structural	Cell Structure	Trafficking	1
myosin	Structural	Cell Structure	Trafficking	2
filamins A and B	Structural	Cell Structure	Trafficking	5
actin	Structural	Cell Structure	Trafficking	11
1,4-alpha-glucan-branching enzyme	Enzyme	Glucose Homeostasis		1
alcohol dehydrogenase [NADP(+)]-like	Enzyme	Glucose Homeostasis		1
c-Cbl associated protein	Regulatory	Glucose Homeostasis		1

fructose-1,6-bisphosphatase	Enzyme	Glucose Homeostasis		1
glucosidase 2	Enzyme	Glucose Homeostasis		1
glycogen debranching enzyme	Enzyme	Glucose Homeostasis		1
glycogen phosphorylase	Enzyme	Glucose Homeostasis		1
glycoside hydrolase	Enzyme	Glucose Homeostasis		1
hexokinase-1	Enzyme	Glucose Homeostasis		1
NADP(+)-dependent isocitrate dehydrogenase	Enzyme	Glucose Homeostasis		1
aldose reductase	Enzyme	Glucose Homeostasis		2
malate dehydrogenase	Enzyme	Glucose Homeostasis		2
phosphoglycerate kinase 1	Enzyme	Glucose Homeostasis		2
glucose-6-phosphate isomerase	Enzyme	Glucose Homeostasis		2
phosphoglucomutase-1	Enzyme	Glucose Homeostasis		2
phosphoenolpyruvate carboxykinase (GTP)	Enzyme	Glucose Homeostasis		3
fructose-bisphosphate aldolase A	Enzyme	Glucose homeostasis		6
enolases	Enzyme	Glucose Homeostasis		14
fibrinogen	Structural	Immune Response and Inflammation		1
fibronectin	Regulatory	Immune Response and Inflammation		1
lipopolysaccharide binding protein (LBP)	Regulatory	Immune Response and Inflammation		1
transferrin	Transport	Immune Response and Inflammation		1
alpha-2-HS-glycoprotein (Fetuin A)	Transport	Immune Response and Inflammation		2
ferritin	Transport	Immune Response and Inflammation		2

alpha-1-antitrypsin	Enzyme	Immune Response and Inflammation		9
complement C1	Immune	Immune Response and Inflammation		1
complement C4	Immune	Immune Response and Inflammation		2
complement C9	Immune	Immune Response and Inflammation		1
complement C3	Immune	Immune Response and Inflammation		5
high mobility group protein B1	Regulatory	Immune Response and Inflammation		1
FK506-binding protein	Immune	Immune Response and Inflammation		1
Lysozyme C	Enzyme	Immune Response and Inflammation		1
monoglyceride lipase	Enzyme	Immune Response and Inflammation		1
Thy-1	Immune	Immune Response and Inflammation		1
neuroleukin	Immune	Immune Response and Inflammation		2
calcineurin B homologous protein 1	Regulatory	Immune Response and Inflammation		2
CD59 glycoprotein	Regulatory	Immune Response and Inflammation		2
alpha-2-macroglobulin	Regulatory	Immune Response and Inflammation		2
cyclophilins	Immune	Immune Response and Inflammation		2
heparin cofactors	Regulatory	Immune Response and Inflammation		2
neutrophil cytosolic factor 3	Enzyme	Immune Response and Inflammation		2
tenascin	Immune	Immune Response and Inflammation		2
dermicidin	Immune	Immune Response and Inflammation		3
B cell antibody	Immune	Immune Response and Inflammation		4
galectins	Immune	Immune Response and Inflammation		4

serpins	Regulatory	Immune Response and Inflammation		5
annexins	Immune	Immune Response and Inflammation		19
Igs	Immune	Immune Response and Inflammation		35
adiponectin	Regulatory	Lipid Metabolism		2
alkyldihydroxyacetonephosphate synthase	Enzyme	Lipid Metabolism		1
acetyl-CoA acetyltransferase	Enzyme	Lipid Metabolism		2
ATP-citrate lyase	Enzyme	Lipid Metabolism		2
1-acylglycerol-3-phosphate O-acyltransferase ABHD6	Enzyme	Lipid Metabolism		1
2-hydroxyphytanoyl-CoA lyase	Enzyme	Lipid Metabolism		1
ATP-citrate synthase	Enzyme	Lipid Metabolism		1
extended synaptotagmin-2	Transport	Lipid Metabolism		1
fatty aldehyde dehydrogenase	Enzyme	Lipid Metabolism		1
GTPase Rab7	Regulatory	Lipid Metabolism		1
lipase	Enzyme	Lipid Metabolism		1
long chain fatty acyl CoA synthetase	Enzyme	Lipid Metabolism		1
fatty acid-binding protein, epidermal	Transport	Lipid Metabolism		2
long-chain-fatty-acid--CoA ligase 1	Enzyme	Lipid Metabolism		2
NADH-cytochrome b5 reductase 3	Enzyme	Lipid Metabolism		2
peroxisomal multifunctional enzyme type 2	Enzyme	Lipid Metabolism		2
trifunctional enzyme	Enzyme	Lipid Metabolism		2
apolipoprotein	Transport	Lipid Metabolism		3

3-ketoacyl-CoA thiolase	Enzyme	Lipid Metabolism		3
glycerol-3-phosphate dehydrogenase [NAD(+)]	Enzyme	Lipid Metabolism		3
peroxisomal acyl-coenzyme A oxidase 1	Enzyme	Lipid Metabolism		3
perilipin-1	Regulatory	Lipid Metabolism		4
3-hydroxyacyl-CoA dehydrogenase	Enzyme	Lipid Metabolism		4
fatty acid synthase	Enzyme	Lipid Metabolism		6
fatty acid-binding protein, adipocyte	Transport	Lipid Metabolism		20
decorin	Structural	Tissue Structure		4
basement membrane-specific heparan sulfate proteoglycan core	Structural	Tissue Structure		6
fibromodulin	Structural	Tissue Structure		1
prolargin	Structural	Tissue Structure		4
nidogen	Structural	Tissue Structure		5
lumican	Structural	Tissue Structure		6
collagen	Structural	Tissue Structure		12
serum albumin	Transport	Transport Protein		26
alpha-1-acid glycoprotein 1	Transport	Transport Protein		2
haptoglobin	Transport	Transport Protein		3
hemopexin	Transport	Transport Protein		5
EH domain-containing protein 2	Transport	Transport Protein		11
fetoprotein	Transport	Transport Protein		1
Acyl-CoA-binding protein	Transport	Transport Protein		1

vitamin D-binding protein	Transport	Transport Protein		2
ceruloplasmin	Transport	Transport Protein		3
serotransferrin	Transport	Transport Protein		3
myoglobin	Transport	Transport Protein		19
hemoglobin	Transport	Transport Protein		36
